# Development and internal validation of a model for predicting cefoperazone/sulbactam-associated coagulation disorders in Chinese inpatients

**DOI:** 10.1186/s40360-024-00761-7

**Published:** 2024-07-12

**Authors:** An Fu, Feng Ge, Yanwei Wang, Haili Guo, Man Zhu, Shu Li, Ao Gao, Chao Li, Jingchuan Lu, Daihong Guo

**Affiliations:** 1 Department of Pharmacy, Medical Supplies Center of Chinese PLA General Hospital, Beijing, 100853 China; 2 Medical School of Chinese PLA, Beijing, 100853 China; 3 The Sixth Medical Center of Chinese PLA General Hospital, Beijing, 100853 China; 4 Faculty of Hepato-Pancreato-Biliary Surgery, Chinese PLA General Hospital, Beijing, 100853 China; 5 College of Pharmacy, Chongqing Medical University, Chongqing, 400016 China

**Keywords:** Adverse drug reaction, Automated monitoring, Cefoperazone/sulbactam, Coagulation disorder, Prediction model, Antibacterial management

## Abstract

**Background and aim:**

The use of cefoperazone/sulbactam (CPZ/SAM) could commonly cause vitamin K-dependent coagulation disorders and even hemorrhage sometimes. However, there is a lack of prediction tools estimating the risk for this. This study aimed at developing and internally validating a model for predicting CPZ/SAM-associated coagulation disorders in Chinese inpatients.

**Methods:**

A case-control study was conducted in 11,092 adult inpatients admitted to a Chinese general hospital between 2020 and 2021 and treated with CPZ/SAM. Patients with CPZ/SAM-associated coagulation disorders were identified through the Adverse Drug Events Active Surveillance and Assessment System-II and subsequent manual evaluation. Controls were selected from eligible patients who didn’t develop coagulation disorders after CPZ/SAM therapy, with a 1:1 propensity score matching. The final predictors were obtained by univariable and multivariable logistic regression analyses. Internal validation and calibration for the model were performed using 1000 bootstrap resamplings.

**Results:**

258 patients were identified as CPZ/SAM-associated coagulation disorders in 2184 patients eligible for inclusions and exclusions and the incidence was 11.8%. A final population of 252 cases and 252 controls was included for model development and validation. Malnutrition (OR = 2.41 (1.56–3.77)), history of recent bleeding (OR = 1.95 (1.32–2.90)), treatment duration (OR = 1.10 (1.07–1.14)), combination with carbapenems (OR = 4.43 (1.85–11.88)), and serum creatinine (OR = 1.01 (1.00–1.01)) were identified as final predictors. The model showed good discrimination, calibration, and clinical practicality, with the validated area under the receiver operating characteristic curve being 0.723 (0.683–0.770).

**Conclusions:**

The model with good performance quantifies the risk for CPZ/SAM-associated coagulation disorders, and may support individual assessment and interventions to mitigate the risk after external validation.

**Supplementary Information:**

The online version contains supplementary material available at 10.1186/s40360-024-00761-7.

## Introduction

Cefoperazone/sulbactam (CPZ/SAM) is a broad-spectrum antibiotic utilized against moderate to severe infections caused by Gram-negative bacteria in most Asian-Pacific countries, including China [1, 2]. One reported side effect is vitamin K-dependent coagulation disorders, which may lead to bleeding and even massive hemorrhage [3, 4]. Supplemental vitamin K should be considered to prevent severe coagulopathy in high-risk patients [4–6], but might be unnecessary in low-risk patients because of uncertain efficacy, side effects, and increased medical costs [5, 7, 8]. Ideally, high-risk patients should be identified as accurately as possible to implement a timely prevention. However, current prediction data are somewhat limited. Previous studies only focused on individual risk factors, such as malnutrition, liver failure, renal failure, and duration of therapy [9–12]. The purpose of this study was to develop and internally validate a risk prediction model for CPZ/SAM-associated coagulation disorders in Chinese inpatients.

## Materials and methods

### Study design and patient selection

This single-center, case-control study was conducted in a Chinese general hospital (Beijing). All patient data including diagnosis, medical orders, laboratory results, surgical procedures, nursing care, and electronic medical records were obtained from the hospital information system (HIS). We screened eligible patients and identified cases suspected of CPZ/SAM-associated coagulation disorders with the help of the Adverse Drug Events Active Surveillance and Assessment System-II (ADE-ASAS-II), an automated monitoring system on adverse drug reaction (ADR) developed by our team [13, 14]. The detailed process was as follows: (i) we performed parameter settings for patient screening in ADE-ASAS-II, and established a connection between ADE-ASAS-II and the HIS; (ii) patients suspected with ADR were automatically identified by ADE-ASAS-II; (iii) then these cases were evaluated separately by two clinical pharmacists based on the Naranjo scale [15] and eligibility criteria, and the inconsistency between the two evaluations was referred to the expert panel for final judgment. The evaluation of ADR was assigned to a probability category from the total score as follows: definite ≥ 9, probable 5 to 8, possible 1 to 4, doubtful ≤ 0, and among them cases with scores ≥ 1 were defined as CPZ/SAM-associated coagulation disorders; (iv) controls were selected through manual chart review from patients eligible for inclusion and exclusion, who didn’t develop coagulation disorders after CPZ/SAM therapy.

Patients aged 18 years or older and hospitalized between 2020 and 2021, who were treated with intravenous CPZ/SAM, were admitted to the study cohort. Patients were excluded with the following conditions: (i) treated with CPZ/SAM for less than two days; (ii) lack of coagulation tests before or after treatment; (iii) diagnosed with terminal liver diseases (including liver cancer, liver cirrhosis and liver failure), hematologic malignancies, sepsis, severe trauma and disseminated intravascular coagulation; (iv) combined with intravenous heparin or oral anticoagulation.

We performed a 1:1 propensity score matching (PSM) between the coagulation disorders group and the control group to minimize confounding bias due to age, gender, length of hospital stays, and cancer. The sample size was calculated based on the 10 EPV (events per variable) principle. Assuming that 15 variables were finally included in the multivariable logistic regression, 150 cases and 150 controls were required at least to ensure the stability of parameter estimates [16]. All methods were carried out following the Transparent Reporting of a multivariable prediction model for Individualized Prognosis Or Diagnosis (TRIPOD) statement [17].

### Data collection and definition

Anonymous patient data were collected from the HIS via ADE-ASAS-II. We considered a CPZ/SAM-associated coagulation disorder based on an increase by more than 25% in prothrombin time (PT), activated partial prothrombin time (APTT), or thrombin time (TT) from baseline [6, 10, 18]. Based on previous similar studies and expert opinions, we identified 23 initial predictors that may be associated with coagulation disorders, including (i) co-morbidities such as malnutrition, hypoproteinemia, and chronic kidney disease [19, 20]; (ii) history of recent surgical procedures or bleeding events [9, 10, 19]; (iii) the duration, daily dose, and cumulative dose of CPZ/SAM therapy [11, 12]; (iv) combinations with other antibacterial agents that may disturb coagulation, including tigecycline, carbapenems, vancomycin, and linezolid [20–22]; (v) baseline laboratory data from the latest blood test within 14 days before CPZ/SAM administration, including liver function indicators (alanine aminotransferase, aspertate aminotransferase, albumin, total bilirubin), renal function indicators (serum creatinine), hemoglobin, platelet count, and coagulation indices (fibrinogen, PT, APTT, TT) [11, 12, 19]. We calculated the geriatric nutrition risk index (GNRI) with height, body weight, and albumin (GNRI = 1.489 × albumin + 41.7 × body weight/ideal body weight (ideal body weight = 22 × height^2^)), and defined GNRI ≤ 91.2 as malnutrition [23, 24]. Chronic kidney disease was identified following the Kidney Disease Improving Global Outcomes (KDIGO) criteria [25].

### Model development and statistical analysis

Missing data were imputed by constructing a multivariable model using the “mice” R package. All initial variables were checked for multicollinearity. After that, univariable and multivariable logistic regression (forward) analyses were performed successively to identify independent risk factors for CPZ/SAM-associated coagulation disorders. Based on these factors, we constructed a nomogram and a web-based probability calculator to visualize the model and facilitate its application. The model was internally validated and calibrated using 1000 bootstrap re-samplings, which could support accurate evaluation of overfitting, especially in small-sample studies [26]. The area under the receiver operating characteristic curve (ROC-AUC) was used to evaluate the discriminatory ability of the model, and the calibration curve was used to analyze the accordance between the predicted probability and the actual observation. Decision curve analysis (DCA) was performed to assess the clinical practicability of the model. In addition, we performed a sensitivity analysis using complete data without missing values. Continuous variables (non-normally distributed) were reported as median (interquartile range (IQR)) and compared between groups by the Mann-Whitney U test. Binary data were presented as absolute numbers (percentages) and compared between groups by the Person’s chi-square test. The statistical analysis was performed using R 4.3.1 and IBM SPSS Statistics 27.0. A *p*-value < 0.05 was considered statistically significant.

## Results

### Patient characteristics

**Fig. 1:**
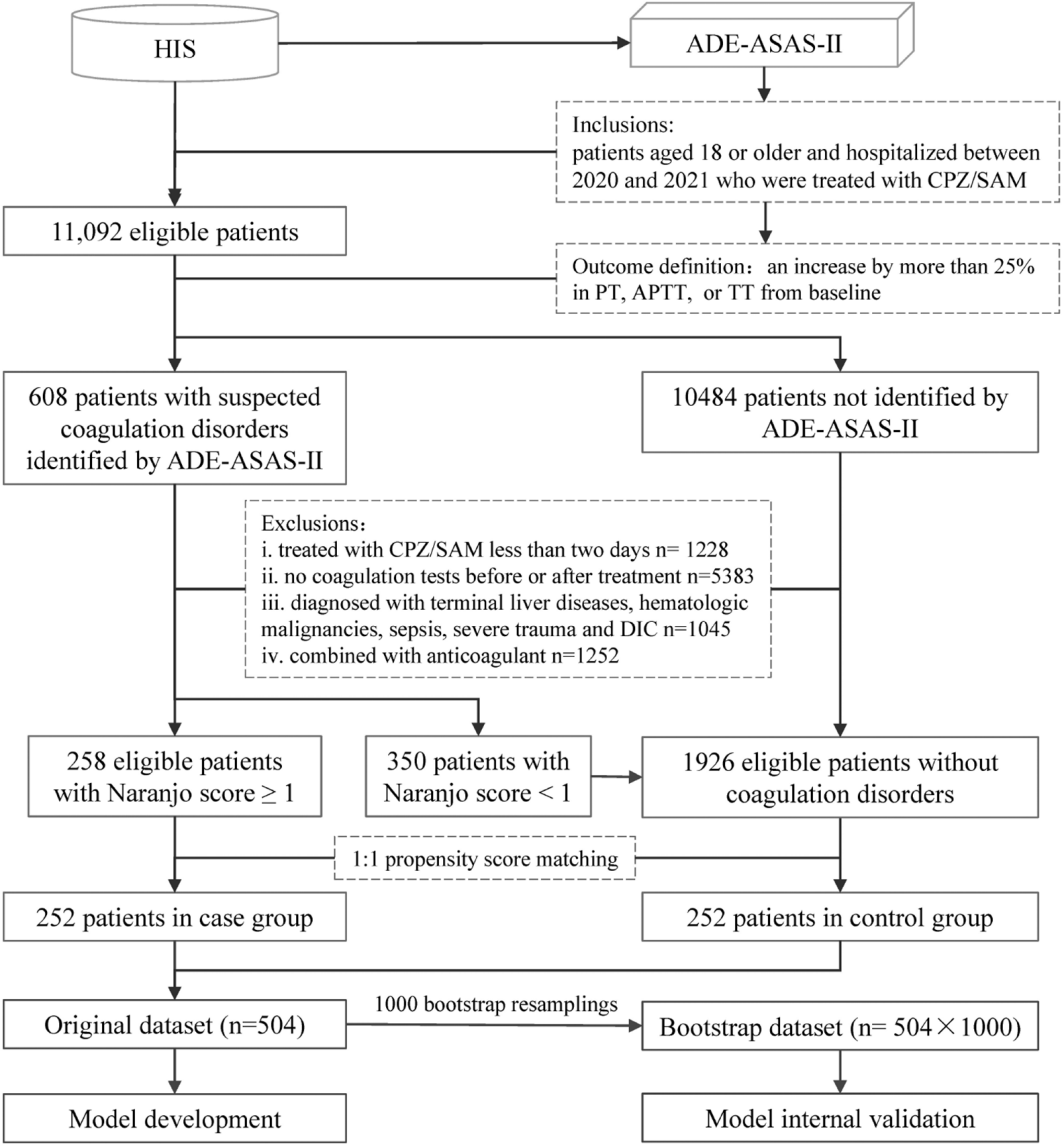
The flow chart of the study. *HIS* hospital information system, *ADE-ASAS-II* Adverse Drug Events Active Surveillance and Assessment System-II, *CPZ/SAM* cefoperazone/sulbactam, *PT* prothrombin time, *APTT* activated partial prothrombin time, *TT* thrombin time, *DIC* disseminated intravascular coagulation

11,092 patients treated with CPZ/SAM were screened by ADE-ASAS-II from 231,705 patients admitted to the hospital between 2020 and 2021. 608 cases were marked with suspected coagulation disorders after CPZ/SAM treatment by ADE-ASAS-II, and among them, 258 were identified by pharmacists through the Naranjo score, including 6 definite cases, 32 probable cases, and 220 possible cases. A total of 1926 eligible patients didn’t develop coagulation disorders after CPZ/SAM treatment and were selected by manual chart review (Fig. 1). The overall incidence of CPZ/SAM-associated coagulation disorders was 11.8% (258/(258 + 1926)), with 4.7% PT prolongations (103 cases), 10.2% APTT prolongations (222 cases) and 1.0% TT prolongations (22 cases). Coagulation disorders occurred mostly within 14 days after the first dose of CPZ/SAM (91%), with a median latency of 4 (2, 8) days. Coagulation returned to normal in 28% of patients (72 cases) after withdrawal or supportive care, with a median duration of 5 (4, 8) days. After PSM, 252 cases each in the case and control groups were obtained for predictors analyses and model development. Baseline characteristics of original data before imputation were shown in Supplementary Table 1.

### Predictor selection

A comparison of baseline characteristics for PSM was shown in Table 1, and there was no difference between groups in these characteristics after PSM (*p* > 0.05). The cumulative dose of CPZ/SAM was excluded from further analysis due to a high correlation with treatment duration (Supplementary Fig. 1). In the analyses of imputed data (Table 2), nine variables were statistically different in univariable analysis (*p* < 0.05). Among them, five predictors including malnutrition (OR = 2.41 (1.56–3.77)), history of recent bleeding (OR = 1.95 (1.32–2.90)), treatment duration (OR = 1.10 (1.07–1.14)), combination with carbapenems (OR = 4.43 (1.85–11.88)), and serum creatinine (OR = 1.01 (1.00–1.01)) were proved to correlate with CPZ/SAM-associated coagulopathy in further multivariable logistic regression (Table 3).

**Table 1:** Baseline characteristics for PSM

Characteristics	Before PSM^a, b^				After PSM^a, b^			
	Total (*n* = 2184)	Coagulation disorders (*n* = 258)	No coagulation disorders (*n* = 1926)	*p* ^c^	Total (*n* = 504)	Case group (*n* = 252)	Control group (*n* = 252)	*p* ^c^
Age, y	59 (47, 70)	66 (53, 81)	58 (46, 69)	< 0.001	66 (54, 79)	66 (53, 80)	66 (54, 79)	0.755
Gender, male	1282 (59%)	165 (64%)	1117 (58%)	< 0.05	325 (64%)	161 (64%)	164 (65%)	0.780
Length of stay, d	13 (9, 20)	23 (15, 43)	12 (9, 19)	< 0.001	22 (15, 40)	23 (15, 42)	22 (14, 37)	0.356
Cancer	748 (34%)	112 (43%)	636 (33%)	< 0.01	218 (43%)	106 (42%)	112 (44%)	0.590

^a^PSM, propensity score matching

^b^Median (interquartile range); absolute numbers (percentages)

^c^Mann-Whitney test; Pearson’s chi-square test

**Table 2:** Univariable analyses of predictors for CPZ/SAM-associated coagulation disorders

Variables	Total (*n* = 504^a^)	Case group (*n* = 252^a^)	Control group (*n* = 252^a^)	Crude OR (95%CI)	*p*
Co-morbidities					
Malnutrition	137 (27%)	91 (36%)	46 (18%)	2.53 (1.68–3.81)	< 0.001
Hypoproteinemia	80 (16%)	48 (19%)	32 (13%)	1.62 (0.99–2.63)	0.053
Chronic kidney disease	70 (14%)	44 (17%)	26 (10%)	1.84 (1.09–3.09)	< 0.05
Medical history					
Recent surgery	113 (22%)	61 (24%)	52 (21%)	1.23 (0.81–1.87)	0.337
Recent bleeding	197 (39%)	112 (44%)	85 (34%)	1.57 (1.10–2.25)	< 0.05
CPZ/SAM therapy					
Duration, d	7 (4, 11)	9 (6, 14)	6 (3, 9)	1.07 (1.03–1.10)	< 0.001
Daily dose, g	6 (6, 6)	6 (6, 6)	6 (6, 6)	1.05 (0.94–1.17)	0.412
Combined antibacterial agents					
Tigecycline	22 (4.4%)	15 (6.0%)	7 (2.8%)	2.22 (0.89–5.53)	0.088
Carbapenems	29 (5.8%)	22 (8.7%)	7 (2.8%)	3.35 (1.40–7.99)	< 0.01
Vancomycin	20 (4.0%)	9 (3.6%)	11 (4.4%)	0.81 (0.33–1.99)	0.649
Linezolid	13 (2.6%)	6 (2.4%)	7 (2.8%)	0.85 (0.28–2.58)	0.779
Baseline laboratory data					
Alanine aminotransferase, U/L	18.1 (11.1, 34.9)	19.0 (11.4, 36.7)	17.2 (10.9, 33.3)	1.00 (1.00–1.00)	0.535
Aspertate aminotransferase, U/L	19.7 (14.5, 32.7)	21.4 (14.9, 37.5)	18.9 (14.3, 29.5)	1.00 (1.00–1.01)	0.103
Albumin, g/L	36.3 (32.5, 40.0)	35.6 (31.5, 39.4)	36.9 (33.2, 40.4)	0.96 (0.93–0.99)	< 0.05
Total bilirubin, µmol/L	11.1 (7.5, 18.8)	11.0 (7.4, 18.8)	11.3 (7.8, 18.7)	1.00 (0.99–1.01)	0.495
Serum creatinine, µmol/L	69.9 (56.6, 89.6)	70.4 (57.0, 95.4)	69.6 (56.0, 85.4)	1.00 (1.00–1.01)	< 0.05
Hemoglobin, g/L	3.80 (3.24, 4.31)	3.70 (3.08, 4.18)	3.93 (3.38, 4.37)	0.72 (0.57–0.90)	< 0.01
Platelet count, 10^9/L	196 (153, 260)	192 (151, 269)	199 (160, 248)	1.00 (1.00–1.00)	0.407
Fibrinogen, g/L	3.6 (2.8, 4.7)	3.5 (2.7, 4.6)	3.6 (2.8, 4.8)	0.98 (0.87–1.10)	0.688
Thrombin time, s	16.3 (15.6, 17.0)	16.4 (15.6, 17.2)	16.3 (15.7, 16.9)	1.05 (0.96–1.15)	0.298
Activated partial prothrombin time, s	34.2 (30.5, 38.9)	34.3 (29.7, 41.1)	34.1 (30.9, 36.9)	1.04 (1.01–1.06)	< 0.01
Prothrombin time, s	13.4 (12.5, 14.5)	13.5 (12.3, 14.7)	13.3 (12.6, 14.2)	1.06 (0.99–1.15)	0.110

^a^Median (interquartile range); absolute numbers (percentages)

*OR* odds ratio, *CI* confidence interval

**Table 3:** The multivariable logistic regression model for CPZ/SAM-associated coagulation disorders

Variables	Coefficient	SE	Z value	Adjusted OR (95%CI)	*p*
Intercept	1.89	0.28	−6.82	0.15 (0.09–0.25)	< 0.001
Malnutrition	0.88	0.23	3.91	2.41 (1.56–3.77)	< 0.001
History of recent bleeding	0.67	0.20	3.32	1.95 (1.32–2.90)	< 0.001
Treatment duration	0.10	0.02	5.51	1.10 (1.07–1.14)	< 0.001
Combination with carbapenems	1.49	0.47	3.18	4.43 (1.85–11.88)	< 0.01
Serum creatinine	0.01	0.00	2.75	1.01 (1.00–1.01)	< 0.01

*SE* standard error, *OR* odds ratio, *CI* confidence interval

### Model development and visualization

**Fig. 2:**
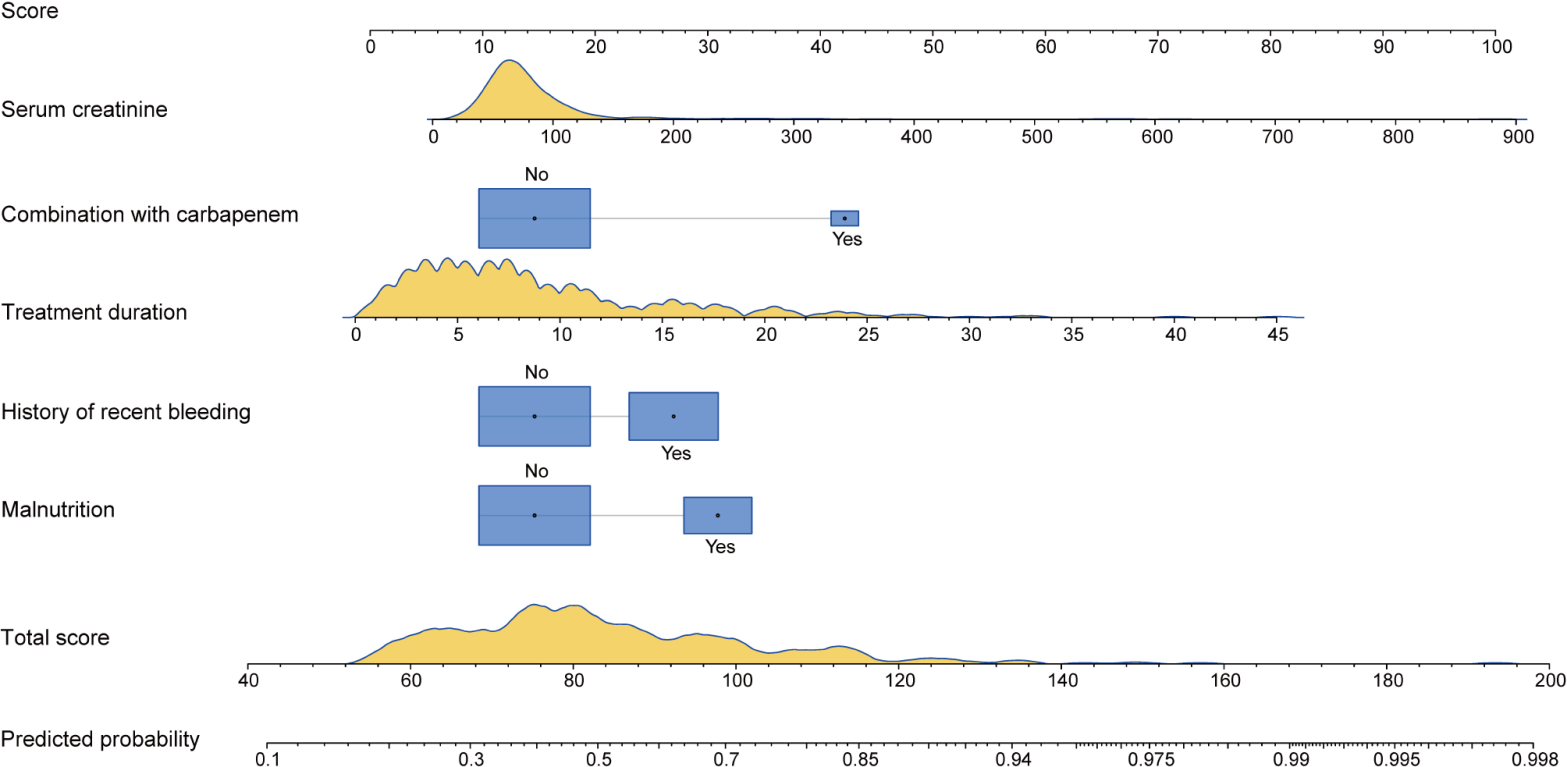
The nomogram for predicting CPZ/SAM-associated coagulation disorders

**Fig. 3:**
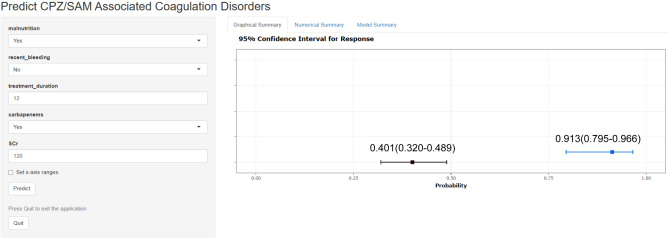
The web-based probability calculator for predicting CPZ/SAM-associated coagulation disorders

Based on the model coefficients in Table 3, we derived the formula for calculating the prediction probability of CPZ/SAM-associated coagulation disorders as *P* = 1/(1 + EXP(−(−1.89 + malnutrition × 0.88 + history of recent bleeding × 0.67 + treatment duration × 0.10 + combination with carbapenems × 1.49 + blood creatinine level × 0.01)). We developed a nomogram to visualize the model. As shown in Fig. 2, the scores corresponding to each predictor in the nomogram (top scale) are summed to obtain the total score (bottom scale), and the probability compatible with the total score is the risk probability for coagulation disorders in patients treated with CPZ/SAM. We also constructed a web-based probability calculator for faster estimation of predicted risk (available for free at: https://coagulopathypredict.shinyapps.io/CPZ-SAM/) (Fig. 3). We demonstrated the application of the nomogram and the probability calculator with two examples. A patient with a history of bleeding and a baseline serum creatinine of 40 µmol/L before a 6-day CPZ/SAM treatment, had a total score of approximately 75 based on the nomogram, and his risk probability of coagulation disorders was 0.401 (0.320–0.489). The other patient, who was diagnosed with malnutrition and received a combination therapy of CPZ/SAM with imipenem-cilastatin for 12 days with a baseline serum creatinine of 120 µmol/L, got a total score of 126 and a predicted probability of coagulation disorders of 0.913 (0.795–0.966) correspondingly.

### Model evaluation and sensitivity analysis

**Fig. 4:**
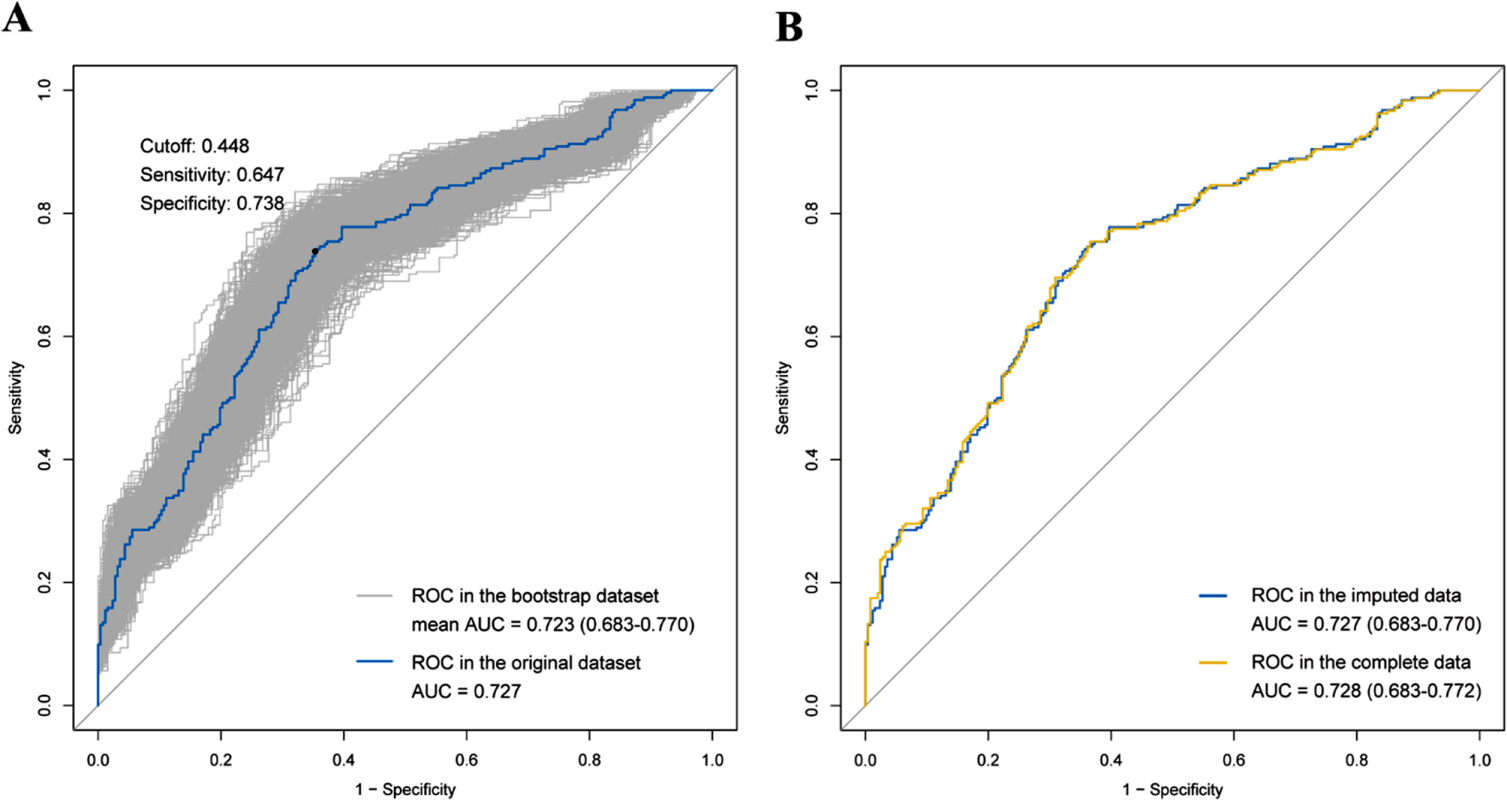
The receiver operating characteristics (ROC) curves of the prediction model (**A**) ROC curves in the original dataset and the bootstrap dataset; (**B**) ROC curves in the imputed data and the complete data

**Fig. 5:**
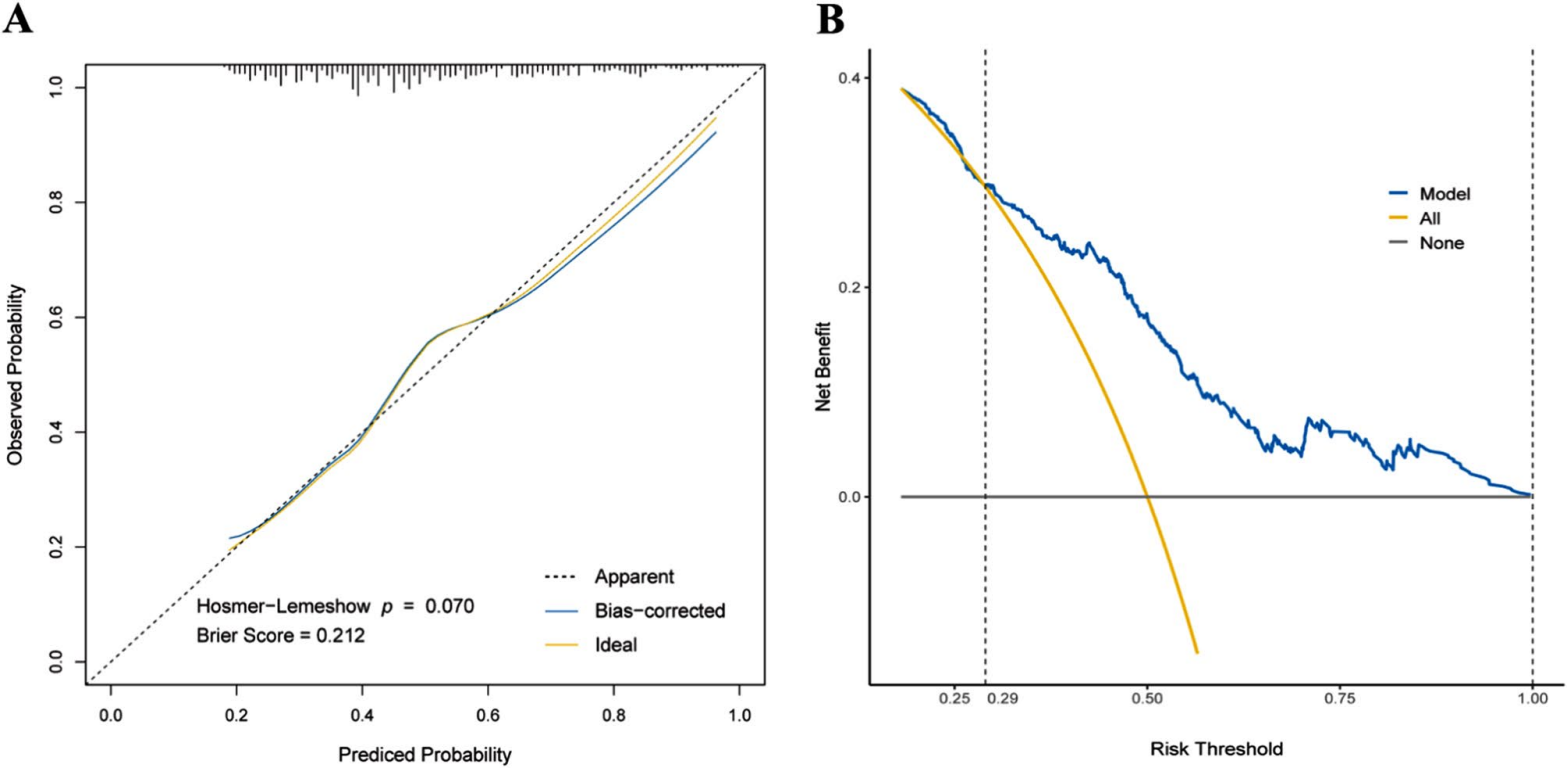
The calibration curve and decision curve analysis (DCA) curve of the prediction model (**A**) the calibration curve; (**B**) the DCA curve

The ROC-AUC of our model is 0.727 in the original dataset and the average ROC-AUC in the bootstrap dataset is 0.723 with a 95% confidence interval of 0.683–0.770, which shows good discrimination and stability (Fig. 4A). The optimal cutoff value of the model in the ROC curve is 0.448, which can be considered as a reference for risk threshold delineation. The calibration curve of the model (Fig. 5A) shows good consistency of observed and predicted values, and the results of the Hosmer-Lemeshow test (*p* = 0.070) and Brier score (0.212) further confirm that the model is well-calibrated. The y-axis of the DCA curve (Fig. 5B) represents the net benefit that patients get, and the x-axis represents the risk threshold of CPZ/SAM-associated coagulopathy. When the threshold probability ranges from 0.3 to 1.0, the application of the model in clinical decision-making may bring more net benefit to patients, compared to the two reference strategies that coagulopathy occurs in all patients with medication (yellow diagonal line) or no coagulopathy occurs (black horizontal line). We additionally performed a sensitivity analysis in complete data without missing values, and as a result, we screened out the same independent risk factors as listed in Table 3 (Supplementary Table 2). The ROC-AUC of the model in the sensitivity analysis was 0.728 (0.683–0.772) (Fig. 4B), getting close to the ROC-AUC of the original model (p for Delong test = 0.978).

## Discussion

The incidence of CPZ/SAM-associated coagulation disorders in this study (11.8%) was similar to that in studies by Wang et al. and Bai et al. (9.2%, 10.6%) [18, 19]. It has also been reported that the prevalence of coagulation disorders induced by CZP/SAM in critically ill patients is higher than 20% [10, 20, 27]. In this study, patients with co-morbidities that may affect coagulation a lot were excluded (including end-stage liver disease, hematological malignancies, sepsis, and disseminated intravascular coagulation) to minimize overestimation of the prevalence [28–31]. The coagulation disorders mostly occurred within 14 days after CPZ/SAM administration and usually improved several days later after withdrawal or symptomatic treatment, which were consistent with those in previous studies [10, 20, 27]. However, fewer patients were tested for blood coagulation during CZP/SAM administration or after the onset of coagulation disorder, which may have led to underdiagnosis or failure to intervene in time and thus worsened the coagulopathy. Considering the high incidence of this ADR, coagulation monitoring should be strengthened in high-risk patients during medication administration, so that abnormalities can be detected as early as possible and targeted intervention can be provided. What’s more, accurate identification of high-risk patients is critical to avoid the aggravation of ADR.

Nomogram models are important clinical tools for disease diagnosis or prognostic assessment. In this study, we developed a model for predicting CPZ/SAM-related coagulation disorders in hospitalized patients, which showed good discrimination, calibration, and clinical practicality in identifying high-risk patients before CPZ/SAM administration. Based on five predictors that were easily available in clinical practice, the model can be used in a variety of healthcare settings. The visualized nomogram and web-based calculator of the model simplified its practical application. Clinicians could determine the risk threshold within a probability range of 0.3–1.0 with the model and implement appropriate early interventions, such as antibacterial regimen adjustment, coagulation monitoring, or vitamin K supplementation, when the patient’s predicted probability rises above this level. Risk factors for CPZ/SAM-associated coagulation disorders have been studied for a long time, but few studies have been able to translate the findings into prediction tools that can be used in clinical practice. Bai et al. [19] recently developed a nomogram, which showed higher discrimination and included five predictors largely different from our study. Differences between these two studies may attribute to different populations and designs.

Among the five predictors evaluated in our model, malnutrition is well-known for its association with coagulation disorders [10, 19, 20, 32]. Vitamin K is crucial for blood clotting and may be deficient in malnourished patients [33]. Additionally, deficiency in serum proteins and other micronutrients may also interfere with coagulation [34, 35]. Nutritional status was usually assessed with the Nutrition Risk Screening (2002) scale in clinical practice [19], but not all patients’ assessments were accurately documented according to chart review. The GNRI is valid but simple for predicting nutrition-related complications, and worthwhile to provide prompt and accurate assessments for patients at risk of malnutrition in multiple healthcare settings [23, 24]. About 84% of sulbactam and 25% of cefoperazone are excreted in the urine and most of the remaining cefoperazone is excreted in the bile, according to the label instructions. As a result of increased biliary excretion of CPZ/SAM in patients with renal insufficiency, the drug inhibition on intestinal synthesis of vitamin K would be enhanced, increasing the risk of coagulation abnormalities [3, 20].

CPZ/SAM-associated coagulation disorders were usually considered time- or dose-dependent [10–12, 19]. However, there were disagreements regarding the optimal risk thresholds for the duration and dosage of CPZ/SAM treatment. Zeng et al. identified high-risk patients with a treatment duration of > 10 days and a daily dosage of > 6 g, but did not state the basis for the delineation [11]; Miao et al. found that the optimal cutoffs were 5 days for the duration of treatment and 48 g for cumulative dose by ROC analysis [12]. CPZ/SAM-associated coagulopathy in this study was associated with treatment duration and cumulative dosage rather than daily dosage, whereas cumulative dose was not analyzed further because of its high correlation with treatment duration. A history of recent bleeding was also a potential risk factor, as confirmed by some previous studies. History of hemorrhagic events was an independent risk factor for cephalosporin-induced bleeding in a study by Chen et al. [9]; patients with coagulation disorders experienced more previous bleeding events in a study by Bai et al. [19], although there was no statistically significant difference in its subsequent multivariable analysis.

In the first study of its kind, we reported that the combination of CPZ/SAM and carbapenem might increase the risk for coagulation disorders. Carbapenems, a kind of broad-spectrum antibiotic, could inhibit the intestinal synthesis of vitamin K as CPZ/SAM do and thus disturb blood clotting [22, 36]. There was no evidence of their synergistic anticoagulant effect, but we considered that these two drugs may burden the coagulation system together and worsen the coagulopathy. Besides, patients receiving combined antibacterial therapy tended to experience more complicated and severe infections than those treated with one antibacterial agent in most cases, and the inflammation activated by infection may contribute to declining coagulation [37].

Some limitations existed inevitably. First, this was a single-center, case-control study characterized by patient selection bias, and for that reason, external validation can’t be reached. Second, stringent admission criteria may have caused a loss of cases with potential CPZ/SAM-associated coagulopathy and a consequent underestimation of its prevalence. Furthermore, the small contribution of the predictor—combinations with carbapenems— may have biased the model estimates. Well-designed multi-center studies with a larger sample size are needed to verify our findings and validate the reproducibility and generalizability of our model.

## Conclusions

This study evaluated the incidence and predictors for CPZ/SAM-associated coagulation disorders, and developed a nomogram model to quantify the risk in a large cohort of Chinese inpatients. The model shows good discrimination, calibration, and clinical utility, and may support individual assessment and interventions of CPZ/SAM-induced coagulopathy after external validation.

### Electronic supplementary material

Supplementary Material 1

Supplementary Material 2

## Data Availability

Data and materials are not publicly available due to privacy and ethical restrictions. The raw de-identified data may be made available upon reasonable request from the corresponding authors.
