# Supplementary figures and images for: Development and internal validation of a model for predicting cefoperazone/sulbactam-associated coagulation disorders in Chinese inpatients
Source: BMC Pharmacol Toxicol. 2024 Jul 12;25:41. doi: 10.1186/s40360-024-00761-7 (PMC11241986; doi:10.1186/s40360-024-00761-7)

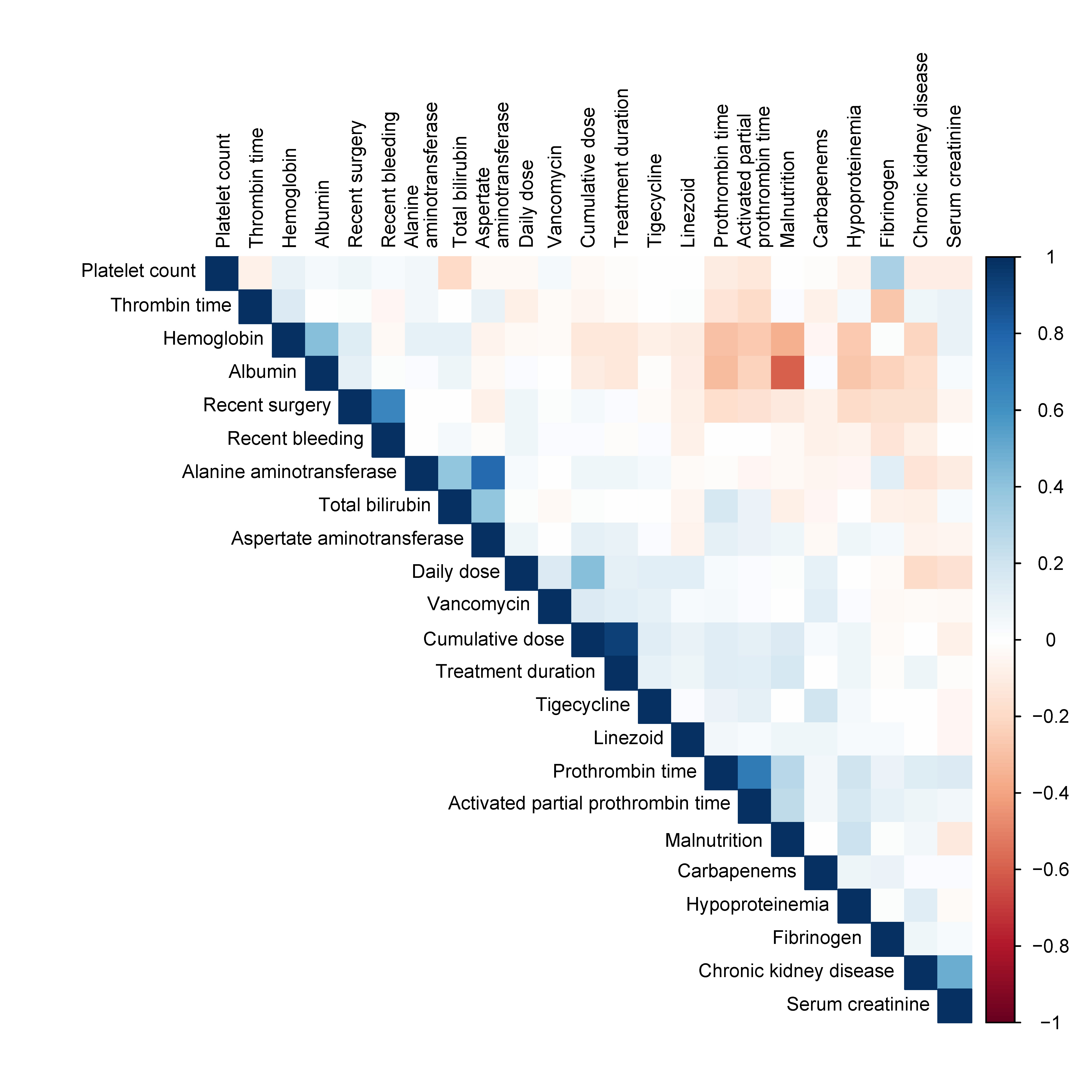


**Supplementary Figure 1**. The multicollinearity between variables

Supplement: Supplementary file 1 — Supplementary Material 1 [file 40360_2024_761_MOESM1_ESM.docx]
