# Supplementary material for: Development and internal validation of a model for predicting cefoperazone/sulbactam-associated coagulation disorders in Chinese inpatients
Source: BMC Pharmacol Toxicol. 2024 Jul 12;25:41. doi: 10.1186/s40360-024-00761-7 (PMC11241986; doi:10.1186/s40360-024-00761-7)
Supplement: Supplementary file 2 — Supplementary Material 2 [file 40360_2024_761_MOESM2_ESM.docx]

Supplementary Table 1. Baseline characteristics

| Characteristics | Total patients (n = 504^1^) |
| --- | --- |
| Age, y | 66 (54, 79) |
| Gender, male | 325 (64%) |
| Length of stay, d | 22 (15, 40) |
| Height, cm | 168 (160, 172) |
| No. of missing cases | 3 |
| Body weight, kg | 64 (57, 73) |
| No. of missing cases | 15 |
| GNRI^2^ | 98 (90, 105) |
| No. of missing cases | 15 |
| Malnutrition | 132 (27%) |
| No. of missing cases | 15 |
| Hypoproteinemia | 80 (16%) |
| Cancer | 218 (43%) |
| Chronic kidney disease | 70 (14%) |
| History of recent surgery | 113 (22%) |
| History of recent bleeding | 197 (39%) |
| Treatment duration, d | 7 (4, 11) |
| Daily dose, g | 6 (6, 6) |
| Cumulative dose, g | 42 (24, 70) |
| Tigecycline | 22 (4.4%) |
| Carbapenems | 29 (5.8%) |
| Vancomycin | 20 (4.0%) |
| Linezolid | 13 (2.6%) |
| Alanine aminotransferase, U/L | 18.1 (11.1, 34.9) |
| Aspertate aminotransferase, U/L | 19.7 (14.5, 32.7) |
| Albumin, g/L | 36.3 (32.5, 40.0) |
| Total bilirubin, μmol/L | 11.1 (7.5, 18.9) |
| No. of missing cases | 3 |
| Serum creatinine, μmol/L | 69.9 (56.6, 89.6) |
| Hemoglobin, g/L | 3.80 (3.24, 4.31) |
| Platelet count, 10^9/L | 196 (153, 260) |
| Fibrinogen, g/L | 3.6 (2.8, 4.7) |
| Thrombin time, s | 16.3 (15.6, 17.0) |
| Activated partial prothrombin time, s | 34.2 (30.5, 38.9) |
| Prothrombin time, s | 13.4 (12.5, 14.5) |
| ^1^ Median (interquartile range); absolute numbers (percentages)  ^2^ GNRI, geriatric nutrition risk index | |

Supplementary Table 2. Univariable and multivariable logistic regression analyses with complete data

| Variables | Total (n=486^1^) | Case group (n=246^1^) | Control group (n=240^1^) | Crude OR (95%CI) | *p* | Adjusted OR (95%CI) | *p* |
| --- | --- | --- | --- | --- | --- | --- | --- |
| Malnutrition | 132 (27%) | 87 (36%) | 45 (18%) | 2.54 (1.67-3.85) | <0.001 | 2.41 (1.54-3.81) | <0.001 |
| Chronic kidney disease | 66 (14%) | 43 (18%) | 23 (9.3%) | 2.12 (1.23-3.64) | <0.01 |  |  |
| History of recent bleeding | 190 (39%) | 107 (45%) | 83 (34%) | 1.58 (1.09-2.28) | <0.05 | 1.95 (1.31-2.92) | <0.01 |
| Treatment duration, d | 7 (4, 11) | 9 (6, 13) | 6 (3, 9) | 1.10 (1.07-1.14) | <0.001 | 1.10 (1.06-1.14) | <0.001 |
| Carbapenems | 28 (5.8%) | 22 (9.2%) | 6 (2.4%) | 4.04 (1.61-10.14) | <0.01 | 5.49 (2.20-15.75) | <0.01 |
| Carbapenems, g/L | 36.2 (32.6, 40.0) | 35.6 (31.6, 39.3) | 36.9 (33.2, 40.4) | 0.96 (0.93-0.99) | <0.05 |  |  |
| Serum creatinine, μmol/L | 69.9 (56.6, 89.1) | 70.1 (56.8, 95.4) | 69.8 (55.9, 85.4) | 1.01 (1.00-1.01) | <0.01 | 1.01 (1.00-1.01) | <0.01 |
| Hemoglobin, g/L | 3.80 (3.24, 4.31) | 3.69 (3.09, 4.14) | 3.95 (3.40, 4.39) | 0.69 (0.54-0.87) | <0.01 |  |  |
| Activated partial prothrombin time, s | 34.1 (30.5, 38.9) | 34.2 (29.7, 41.0) | 34.1 (30.9, 36.9) | 1.04 (1.01-1.06) | <0.01 |  |  |
| ^1^ Median (interquartile range); absolute numbers (percentages)  OR, odds ratio; CI, confidence interval | | | | | | | |
